# Myosteatosis and bone marrow adiposity are not associated among postmenopausal women with fragility fractures

**DOI:** 10.3389/fendo.2023.1178464

**Published:** 2023-06-19

**Authors:** Sammy Badr, Héloïse Dapvril, Daniela Lombardo, Huda Khizindar, Claire Martin, Bernard Cortet, Anne Cotten, Julien Paccou

**Affiliations:** ^1^ University of Lille, MABlab ULR 4490, Lille, France; ^2^ CHU Lille, Department of Radiology and Musculoskeletal Imaging, Lille, France; ^3^ CHU Lille, Department of Rheumatology, Lille, France; ^4^ University of Lille, CHU Lille, ULR 2694 - METRICS: Évaluation des technologies de santé et des pratiques médicales, Lille, France; ^5^ CHU Lille, Department of Biostatistics, Lille, France

**Keywords:** bone-fat interactions, bone-muscle interactions, osteoporosis, menopause, MRI, myosteatosis, bone marrow adiposity

## Abstract

**Objectives:**

Although paravertebral intramuscular fatty infiltration (known as myosteatosis) following a vertebral fracture is well-known, scarce data are available regarding interactions between muscle, bone, and other fat depots. Based on a homogeneous cohort comprising postmenopausal women with or without a history of fragility fracture, we aimed to better depict the interrelationship between myosteatosis and bone marrow adiposity (BMA).

**Methods:**

102 postmenopausal women were included, 56 of whom had a fragility fracture. Mean proton density fat fraction (PDFF) was measured in the psoas (PDFF_Psoas_) and paravertebral (PDFF_Paravertebral_) muscles at the lumbar level, as well as in the lumbar spine and non-dominant hip using chemical shift encoding-based water-fat imaging. Visceral adipose tissue (VAT) and total body fat (TBF) were assessed using dual X-ray absorptiometry. Statistical models were adjusted for age, weight, height (all comparisons), and bone mineral density (when considering BMA).

**Results:**

PDFF in the psoas and paravertebral muscles was higher in the fracture group compared to controls even after adjustment for age, weight, and height (PDFF_Psoas_ = 17.1 ± 6.1% versus 13.5 ± 4.9%, p=0.004; PDFF_Paravertebral_ = 34.4 ± 13.6% versus 24.9 ± 8.8%, p=0.002). Higher PDFF_Paravertebral_ was associated with lower PDFF at the lumbar spine (*β* = -6.80 ± 2.85, p=0.022) among controls but not in the fracture group. In both groups, a significant relationship between higher PDFF_Psoas_ and higher VAT was observed (*β* = 20.27 ± 9.62, p=0.040 in the fracture group, and *β* = 37.49 ± 8.65, p<0.001 in the control group). Although solely observed among controls, a similar relationship was observed between PDFF_Paravertebral_ and TBF (*β* = 6.57 ± 1.80, p<0.001). No significant association was observed between BMA and other fat depots.

**Conclusion:**

Myosteatosis is not associated with BMA among postmenopausal women with fragility fractures. Whereas myosteatosis was associated with other fat depots, BMA appears uniquely regulated.

## 1 Introduction

The aging process is characterized by an increase in total body fat (TBF) mass and a change in body composition with fat distribution resulting in increased ectopic fat accumulations, notably in the musculoskeletal system, which includes bones and muscles (1). Skeletal muscle fat infiltration is known as myosteatosis, while the fat infiltration within the bone marrow is called marrow adiposity (2, 3). Myosteatosis is recognized to correlate with muscle mass, strength, and function negatively but is not synonymous with sarcopenia (2). Marrow adiposity is identified to correlate with bone mass negatively and may be driving bone loss —at least in part— and contributing to osteoporosis (3).

An important area of investigation would be considering the relationship between myosteatosis and marrow adiposity to understand better the muscle-bone-fat crosstalk in aging (4). Beyond the mechanical interaction between bone and muscle, several signaling factors produced by the muscle (myokines), bone (osteokines), and fat (adipokines) have emerged as potential mediators of biochemical/molecular interactions (5). One representative illustration would be the secretion of myostatin. Observed in states of muscle disuse, this muscle-derived cytokine may promote bone-resorbing cells and negatively impact bone remodeling. In patients with visceral adipose tissue accumulation, the upregulation of interleukine 6 (IL-6) induced by the hyperleptinemia is inversely correlated with bone mineral density (BMD) (5). The combination of these situations, frequently encountered in clinical practice, would foster bone fragility. Further understanding of interactions could lead to possible preventive and therapeutic approaches for mitigating osteoporosis and even the commonly co-occurring sarcopenia (6).

Limited data are available on the relationship between myosteatosis and marrow adiposity. In a cross-sectional study, Wong et al. found that fat within the bone and muscle of the mid-leg were related in postmenopausal women. This relationship was modulated by osteoporosis using the WHO definition (7). In a study by Burian et al., marrow adiposity and myosteatosis were not significantly associated with each other at the lumbosacral spine level in 103 healthy volunteers (21–77 years) (8). These results support the notion that fat may be preferentially accumulating within muscle and bone only in patients with osteoporosis.

Then, additional research is needed to confirm whether this relationship depends on the presence of fragility fractures rather than osteoporosis in postmenopausal women (7). Comparison across previous studies is tricky due to the non-standardized assessment of myosteatosis and marrow adiposity, including analysis from different regions of the body and the use of various tools such as peripheral quantitative computed tomography (pQCT) (7) or Magnetic Resonance Imaging (MRI) scans (9).

This study first aimed to explore the relationship between myosteatosis and marrow adiposity in postmenopausal women with and without fragility fracture. The relationships between myosteatosis, marrow adiposity, and other fat depots were explored in those with and without fragility fractures.

## 2 Patients and methods

### 2.1 Study design

This study is an ancillary work based on the ADIMOS cohort (Clinical Gov NCT03219125), including postmenopausal women enrolled by the Department of Rheumatology at Lille University Hospital (France) between October 2018 and June 2021 (10). Two groups were compared. *Exposed participants* were postmenopausal women with a fragility fracture that occurred within the 12 previous months. In contrast, *Non-exposed participants* corresponded to postmenopausal women with osteoarthritis without any fragility fracture history. The study protocol was approved by the local Institutional Review Board (2017-A00472-51), and the study procedures complied with the ethical standards of the relevant institutional and national Human Experimentation Ethics Committees. All patients provided their written informed consent.

### 2.2 Study population

#### 2.2.1 Fractured group

*Inclusion criteria* were: postmenopausal women between 50-90 years old, living in France, and seen by the Fracture Liaison Service at Lille University Hospital for a fragility fracture, defined as a fracture in response to low-energy trauma (e.g., a fall from standing height). Fragility fractures were hip, vertebral, proximal humerus, pelvis, ribs, or forearm/wrist fractures. Eligible patients must be included and interviewed within 12 months of diagnosis of the fracture event.

*Exclusion criteria* were: incomplete MRI protocol (e.g., absence of acquisition allowing the exploration of the paravertebral muscles); implants that were contraindicated for the MRI examination; Implants that might create a health risk or other problem during an MRI scan, i.e., 1) cardiac pacemaker or implantable defibrillator, 2) catheter that has metal components that may pose a risk of a burn injury, 3) a ferromagnetic metal clip placed to prevent bleeding from an intracranial aneurysm, 4) an implanted medication pump (such as that used to deliver insulin or a pain-relieving drug), and 5) a cochlear implant; body mass index (BMI) > 38 kg/m², weight > 140 kg; chronic kidney disease with calculated creatinine clearance < 30 mL/min; disease known to affect bone metabolism; current use of medications known to affect bone mineral density (BMD), including oral glucocorticoids, treatments for osteoporosis (bisphosphonates, raloxifene, teriparatide, or parathyroid hormone), menopausal hormone therapy.

#### 2.2.2 Non-fractured group

*Inclusion criteria* were: postmenopausal women between 50-90 years old, living in France, and seen by the Department of Rheumatology at Lille University Hospital for osteoarthritis (hips, knees, hands, or spine). Non-fractured participants were eligible for the study if they reported no history of a fragility fracture after age 40.

*Exclusion criteria* were similar to those required in the “fracture group.”

### 2.3 Demographic characteristics

All participants underwent a complete musculoskeletal physical examination by an experienced rheumatologist with expertise in managing patients with osteoporosis (JP, 13 years of experience). Inclusion and exclusion criteria were reviewed. Other comorbidities were looked for (e.g., type 2 diabetes mellitus, chronic pulmonary disease, cardiovascular events), and the Charlson Comorbidity Index (CCI) was calculated. Lifestyle characteristics were assessed using the leisure time activity score. Medication data and relevant disorders were collected from all patients.

### 2.4 Bone marrow adiposity and myosteatosis quantification

#### 2.4.1 MRI Acquisition

All subjects underwent an MRI examination on a 3 Tesla system (Ingenia; Philips Healthcare, Best, the Netherlands) using a built-in 12-channel posterior body coil and a 16-channel anterior coil under the supervision of a senior musculoskeletal radiologist (SB, 11 years). Patients were positioned head-first in a supine position. After a conventional assessment using T1 and T2-weighted 2-point Dixon turbo spin acquisitions, bone marrow adiposity and lumbar paravertebral myosteatosis quantification were achieved using six-echo three-dimensional gradient-echo sequences (mDixon-Quant; Philips Healthcare, Best, the Netherlands). Subsequently, a chemical shift encoding-based water-fat separation at the lumbar spine (sagittal), the non-dominant hip (coronal oblique), and the lumbar paravertebral region (axial) could be performed. At the lumbar spine, imaging parameters were: repetition time (TR)/echo time (TE)/ΔTE: 11/1.43/1.1 ms; field of view (FOV): 220 × 220 mm; voxel size: 1.8 × 1.8 mm; slice thickness: 3 mm; number of excitations: 1; no SENSE acceleration; fold-over direction: foot-head; bandwidth: 1563 Hz and scan time: 1 min 41 s. At the hip, MR parameters were: TR/TE/ΔTE: 11/1.13/1.0 ms; FOV: 354 × 354 mm; voxel size: 1.8 × 1.8 mm; slice thickness: 3 mm; number of excitations: 1; no SENSE acceleration; fold-over direction: right-left; bandwidth: 1724 Hz and scan time: 1 min 25 s. For the exploration of the lumbar paravertebral region, MR parameters were: TR/TE/ΔTE: 5.7/1.0/0.7 ms; FOV: 400 × 350 mm; voxel size: 2.5 × 2.5 mm; slice thickness: 6 mm; gap: 3 mm; number of excitations: 1; no SENSE acceleration; fold-over direction: right-left; bandwidth: 2367 Hz and scan time: 16 s (breath-hold). A low flip angle of 3° was used in all situations to minimize T1 bias (11). Offline reconstructions computed proton density fat fraction maps (PDFF; the ratio of the fat signal over the fat and water signals) using a precalibrated seven-peak liver fat spectrum and a single T2*-correction.

#### 2.4.2 Bone marrow adiposity quantification

**Figure 1:**
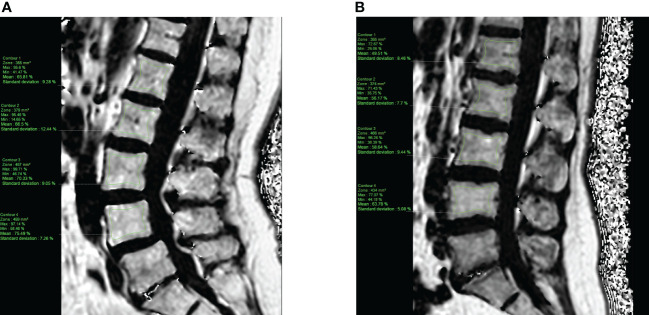
PDFF map of the lumbar spine computed from a T1-weighted multi-echo gradient echo sequence (mDixon-Quant) acquired in the sagittal plane, from a 63-year old control **(A)** and a 50-year old case (fragility fracture of the wrist, **(B)** post-menopausal women. Manually segmented ROIs (*green*) were placed in the vertebral bodies of L1 to L4, avoiding the immediate subchondral bone, the cortical bone and the basivertebral vein.

**Figure 2:**
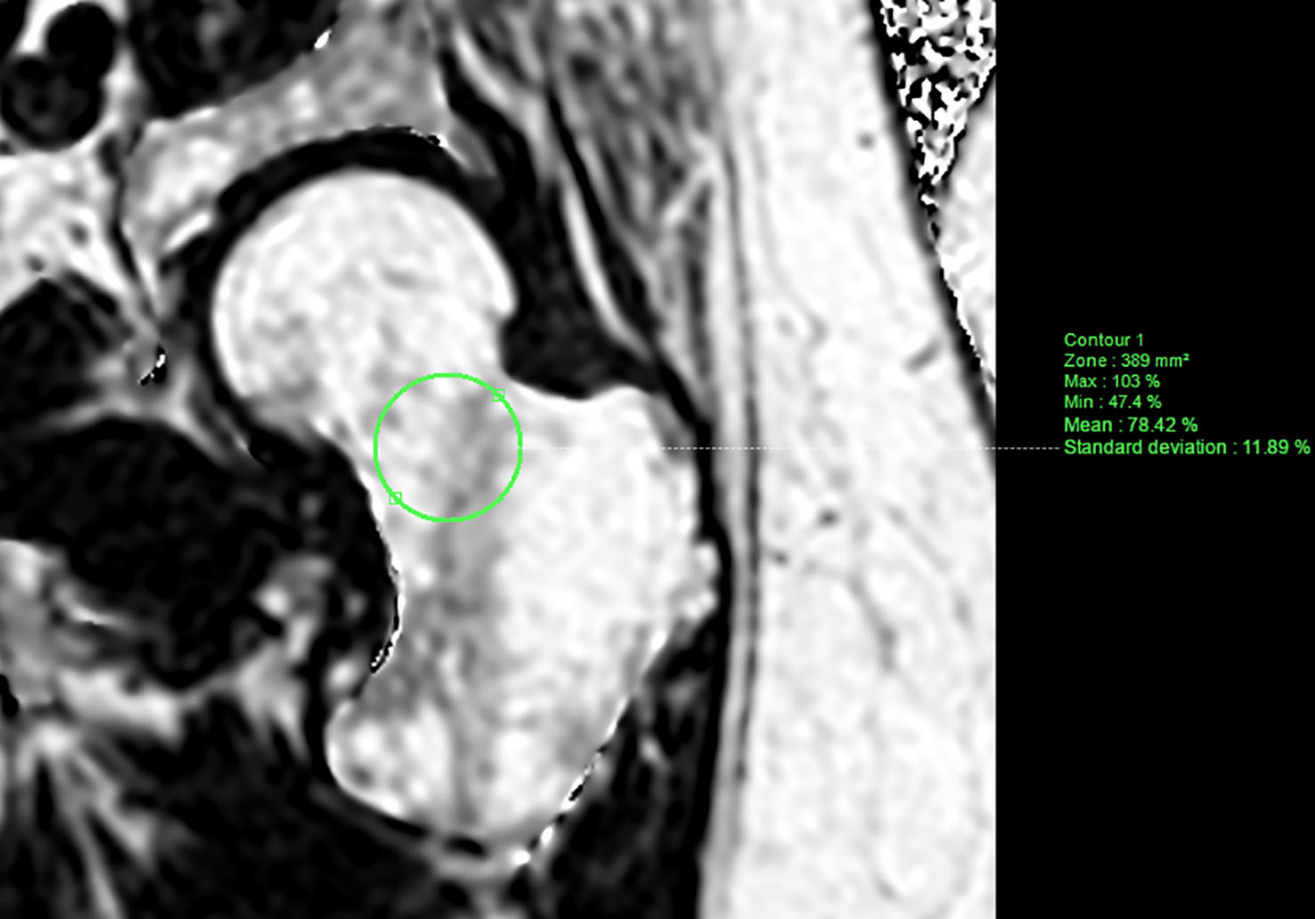
PDFF map of the left (non-dominant) hip computed from a T1-weighted multi-echo gradient echo sequence (mDixon-Quant) acquired in a coronal oblique plane (along the femoral neck axis), from a 51-year old case. Manually segmented ROI (*green*) was placed in the femoral neck avoiding the cortical bone.

Data for bone marrow adiposity quantification were extracted from the main ADIMOS study. As a reminder, the procedure was performed according to the following key points: (1) MRI acquisitions of each subject were reviewed by a senior musculoskeletal radiologist (SB, 11 years) on a dedicated workstation using IntelliSpace Portal (Philips Healthcare; Best, the Netherlands); (2) a morphological assessment was first performed to consider any transitional anomaly, severe degenerative changes, or bone marrow-replacing lesions at the hip or lumbar spine; (3) the three most central slices were chosen at the lumbar spine, based on the PDFF maps computed from mDixon-Quant acquisitions; (4) A polygonal region of interest (ROI) was drawn in the vertebral body of L1 to L4, avoiding fractured vertebrae, the immediate subchondral bone, bone marrow-replacing lesions, severe degenerative changes, and the basivertebral vein. **Figure 1** shows two participants’ PDFF maps of the lumbar spine, with the corresponding segmentation. Similarly, an ROI was drawn in the femoral neck based on the three most central slices of the coronal oblique mDixon-Quant acquisition of the non-dominant hip. **Figure 2** shows a PDFF map of the non-dominant hip of one participant.

#### 2.4.3 Myosteatosis quantification

**Figure 3:**
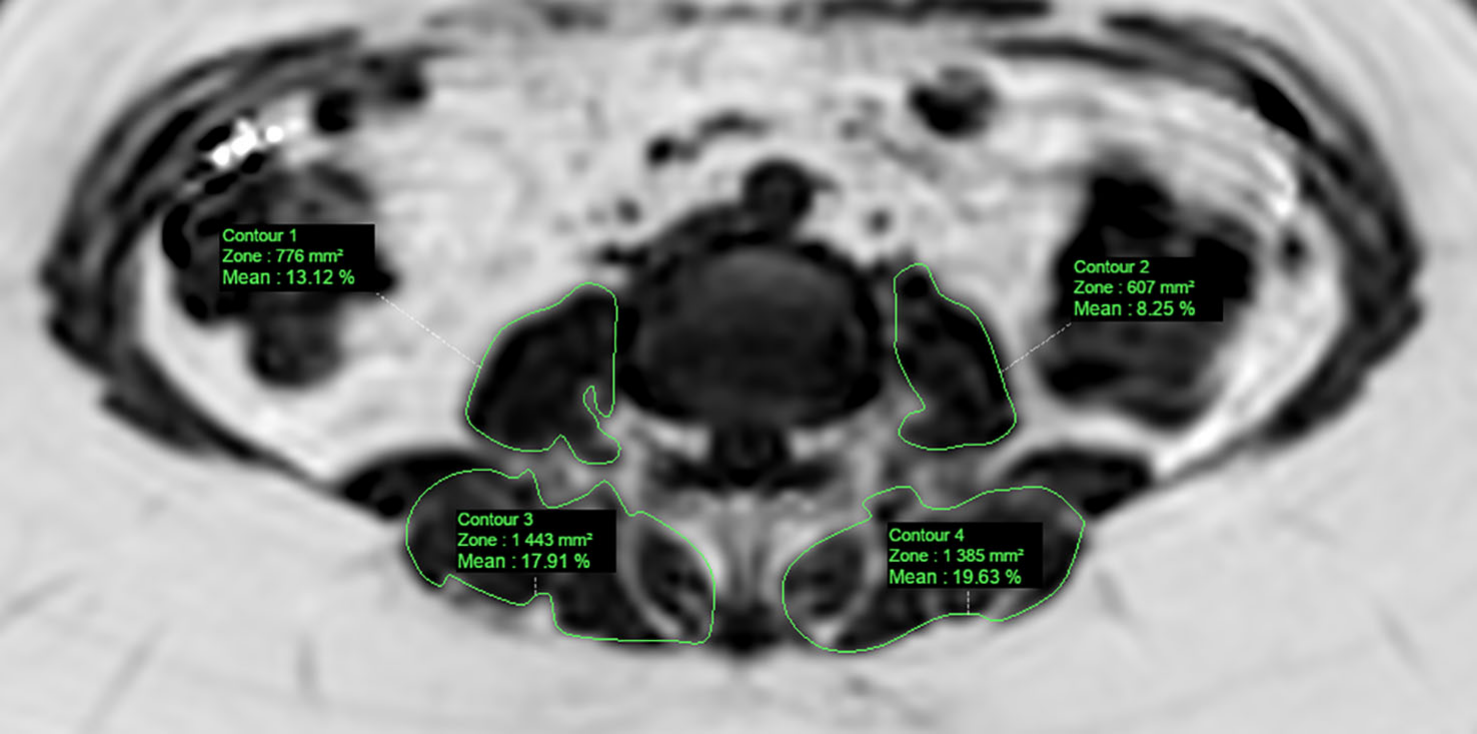
PDFF map of the paravertebral muscles computed from a T1-weighted multi-echo gradient echo sequence (mDixon-Quant) acquired in an axial oblique plane (L3-L4 level), from a 58-year old case. Manually segmented ROI (*green*) was placed in the psoas and paravertebral (multifidus+erector spinae) muscles.

Similarly, MRI acquisitions of the paravertebral lumbar region were segmented by a musculoskeletal radiologist (HD, four years of experience) on a dedicated workstation using IntelliSpace Portal (Philips Healthcare; Best, the Netherlands). Freehand ROI was drawn based on visible muscle boundaries and excluding epimuscular fat, as described by the 2^nd^ method reported by Berry et al. (12). The psoas and paravertebral (combination of the erector spinae and multifidus muscles) muscles were segmented in the axial plane using the computed PDFF maps from the breath-hold mDixon-Quant acquisition. Averaged on both sides and at L2-L3, L3-L4, and L4-L5 levels, the extracted muscle features included the cross-sectional area (CSA, mm^2^), PDFF (%) and subsequent contractile mass index (CMI, mm^2^; CMI = (1-PDFF) × CSA) (9, 13). **Figure 3** shows a PDFF map of one participant’s lumbar paravertebral region with concurrent segmentation.

#### 2.4.4 Repeatability

To assess the MR analysis’s inter-observer agreement, a random subset of 30 subjects (15 participants from each group) was selected using the same tools and segmentation strategy. PDFF values at the lumbar spine (average of the L1-L4 vertebrae), the femoral neck of the non-dominant hip, the psoas and paravertebral muscles were assessed by two independent musculoskeletal radiologists (SB and HK for the spine and the hip; SB and HD for the psoas and paravertebral muscles). For intra-observer agreement, a senior musculoskeletal radiologist (SB) assessed the same subset of subjects. A new segmentation was repeated three months later (for the spine, the femoral neck, the psoas, and the paravertebral muscles).

### 2.5 Bone mineral density and body composition

Bone mineral density (BMD) was measured at the lumbar spine (L1–L4) and the non-dominant hip by dual-energy X-ray absorptiometry (Hologic Discovery A; Hologic, Marlborough, Marlborough, USA). The machine was calibrated daily, and quality-assurance tests were carried out daily and weekly. WHO criteria were used to define osteoporosis and osteopenia based on BMD (T-score ≤ −2.5 and T-score between −1.0 and −2.5, respectively). Body composition derived from DXA acquisitions was obtained in all patients. It comprised BMI calculation (kg/m²) from the subject’s height and weight, as well as an estimate of the visceral adipose tissue (VAT, cm^2^), total body fat (TBF, %), and total and appendicular lean masses (kg).

### 2.6 Laboratory variables

After fasting for at least 8 hours, blood samples were obtained. Routine assays assessed creatinine and high-sensitivity C-reactive protein (hs-CRP). The estimated glomerular filtration rate (GFR) was calculated using the CKD formula (mL/min). Intact parathyroid hormone (PTH) was measured using a chemoluminescent immunoassay on an Architect automatic analyzer (Abbott Laboratories; Chicago, Illinois, USA). 25-hydroxyvitamin D was measured by a competitive chemoluminescent immunoassay on an IDS-iSYS instrument (IDS; Pouilly-en-Auxois, France). Procollagen 1 intact N-terminal (P1NP) and serum cross laps (CTX) were measured by chemiluminescence assay using the IDS-iSYS Multi-Discipline Automated Analyzer (Immunodiagnostic Systems, Inc., Fountain Hills, AZ).

### 2.7 Study objectives

#### 2.7.1 Primary objective

Relationship between Myosteatosis and Bone marrow adiposity in postmenopausal women with a history of fragility fracture.

#### 2.7.2 Secondary objectives

Relationship between Myosteatosis and Bone marrow adiposity in postmenopausal women without a history of fragility fracture.

Relationship between Myosteatosis and Body composition (VAT and TBF) in postmenopausal women with and without a history of fragility fracture.

Relationship between Bone marrow adiposity and Body composition (VAT and TBF) in postmenopausal women with and without a history of fragility fracture.

### 2.8 Statistical analysis

Qualitative variables are reported as frequency (percentage). Continuous variables are reported as mean ± standard deviation in case of a normal distribution or as median [25th to 75th percentiles] otherwise. The normality of distributions was assessed using histograms and using the Shapiro-Wilk test. At baseline, the patient’s general characteristics and biochemistry results were compared between the two groups (exposed/non-exposed) using Chi-Square or Fisher exact test for categorical variables and Student t-test in case of a normal distribution or Mann-Whitney U test otherwise for continuous variables. Postmenopausal women’s paravertebral and psoas muscle features were compared between the two groups using analysis of covariance without and with adjustment for age, weight, and height. The relationship between myosteatosis and bone marrow adiposity, myosteatosis and VAT, myosteatosis and TBF, bone marrow adiposity and VAT, and bone marrow adiposity and TBF were investigated with and without adjustment for predefined confounding factors in fractured/non-fractured group separately, using analyses of variance. For each model, the normality of residuals was checked graphically. We performed two different adjusted models. The first was only adjusted for age. The second was adjusted for age, weight, height, and adding the bone mineral density according to the studied bone marrow adiposity. In each group, the intra- and inter-observer agreement for postmenopausal women’s paravertebral and psoas muscle features was calculated using the concordance correlation coefficient and its 95% confidence interval (14). Values were interpreted using Landis & Koch classification (0, disagree; 0-0.20, very low agreement; 0.21-0.40, low agreement; 0.41-0.60, moderate agreement; 0.61-0.80, important agreement; 0.81-1, almost perfect agreement). All statistical tests were done at the two-tailed α-level of 0.05 using the SAS software version 9.4 (SAS Institute, Cary, NC).

## 3 Results

### 3.1 Baseline characteristics

**Table 1** shows the baseline characteristics. Among the initial 199 participants, 97 postmenopausal women were excluded from the original ADIMOS cohort (paravertebral acquisitions missing or insufficient quality due to respiratory artifacts). In the remaining 102 postmenopausal women with no recent use of bone-active medication included in this ancillary study, non-fractured women (n=46) were significantly younger (p=0.001), taller (p=0.010), and heavier (p=0.032) than fractured participants (n=56). Comorbidities and osteoporosis risk factors were comparable in both groups. In parallel, the CCI was lower in the non-fractured group (p<0.001). Regarding biochemistry results, non-fractured postmenopausal women had significantly lower PINP (p<0.001) than fractured patients. In the fractured group (n=56), we included 28 women with at least one vertebral fracture (median [min-max]: 1 (1–4)), 28 women with non-vertebral fractures (11 forearm/wrist fractures, 9 hip fractures, 5 pelvis fractures, and 3 proximal humerus fractures), and 31 (55.4%) had a history of osteoporotic fractures.

**Table 1:** Patients’ general characteristics and biochemistry results at baseline.

	N	Fracture (n=56)	N	No fracture (n=46)	p-value
Age [years]	56	68.5 (61.5 to 77.0)	46	62.5 (56.0 to 66.0)	**0.001**
Weight [kg]	56	65.5 (57.5 to 72.5)	46	71.5 (61.0 to 81.0)	**0.032**
Height [cm]	56	159.0 (154.5 to 164.0)	46	162.5 (158.0 to 167.0)	**0.010**
BMI [kg/m²]	56	25.2 (22.1 to 29.7)	46	26.6 (23.4 to 29.8)	0.22
Leisure time activity (score 0-15)	56	9.0 (7.0 to 11.0)	46	9.0 (7.0 to 11.0)	0.88
COMORBIDITIES					
Type 2 diabetes mellitus	56	6 (10.7)	46	5 (10.9)	1.00
Chronic pulmonary disease	56	5 (8.9)	46	2 (4.3)	NA
Stroke or transient ischemic attack	56	4 (7.1)	46	1 (2.2)	NA
Charlson Comorbidity Index	56	3.0 (2.0 to 5.0)	46	2.0 (0.0 to 3.0)	**<0.001**
OSTEOPOROSIS RISK FACTORS					
Excessive alcohol consumption	56	5 (8.9)	46	3 (6.5)	0.73
Current smoking	56	7 (12.5)	46	4 (8.7)	0.75
Family history of hip fracture	56	6 (10.7)	46	9 (19.6)	0.21
Previous use of corticosteroids	56	4 (7.1)	46	1 (2.2)	NA
BIOCHEMISTRY RESULTS					
Hs-CRP [mg/L]	56	3.0 (3.0 to 8.5)	46	3.0 (3.0 to 4.0)	0.20
25(OH) vitamin D [ng/mL]	56	29.5 (23.0 to 36.0)	46	27.5 (19.0 to 33.0)	0.31
Serum PTH [pg/mL]	56	43.0 (30.5 to 57.0)	46	47.5 (38.0 to 60.0)	0.31
Creatinine [µmol/L]	56	62.0 (53.0 to 75.5)	46	62.0 (53.0 to 71.0)	0.77
HbA1c [%]	56	5.5 (5.4 to 5.9)	46	5.6 (5.3 to 5.7)	0.74
P1NP [ng/mL]	54^a^	72.5 (54.0 to 99.0)	45^a^	54.0 (40.0 to 69.0)	**<0.001**
CTX [pmol/L]	56	3777 (2415 to 4986)	46	2902 (1921 to 4322)	0.14
BODY COMPOSITION					
Total body fat mass [kg]	54^b^	27.9 (21.7 to 35.2)	46	32.1 (24.4 to 40.7)	0.074
Visceral adipose tissue [cm^2^]	54^b^	140.5 (81.0 to 201.0)	46	148.5 (87.0 to 211.0)	0.63
Total lean mass [kg]	54^b^	33.8 (31.2 to 36.1)	46	36.5 (33.4 to 39.1)	**0.038**
Appendicular lean mass [kg]	54^b^	13.0 (11.2 to 14.5)	46	13.9 (12.5 to 15.0)	0.069
BONE MINERAL DENSITY					
T-Score at the lumbar spine	56	-1.4 ± 1.4	46	-0.8 ± 1.4	**0.040**
T-Score at the femoral neck	53^c^	-1.9 ± 1.0	46	-1.1 ± 1.1	**<0.001**
T-Score at the total hip	53^c^	-1.3 ± 0.9	46	-0.5 ± 1.2	**<0.001**
BMD lumbar spine (g/cm²)	56	0.86 ± 0.15	46	0.92 ± 0.15	**0.038**
BMD femoral neck (g/cm²)	53^c^	0.63 ± 0.12	46	0.73 ± 0.13	**<0.001**
BMD total hip (g/cm²)	53^c^	0.77 ± 0.12	46	0.87 ± 0.16	**<0.001**
BONE MARROW ADIPOSITY					
PDFF lumbar spine (%)	56	58.34 ± 10.18	46	54.93 ± 9.82	0.091
PDFF femoral neck (%)	52^d^	81.39 ± 8.24	46	81.76 ± 7.71	0.82

Values are expressed as numbers (%), as mean ± SD or as median (IQR).

NA, Not Applicable; SD, Standard Deviation; IQR, Interquartile Range; PTH, parathyroid hormone; P1NP, procollagen type 1 N-terminal propeptide; CTX, collagen type 1 cross-linked C-telopeptide; hs-CRP, high-sensitivity C-reactive protein; TIA, transient ischemic attack; BMD, bone mineral density; PDFF, proton density fat fraction.

a P1NP measurements were not available in 3 patients (fractured group: 2; non-fractured group: 1).

b Body composition measurements were not available in 2 patients.

c Hip BMD measurements were not available in 3 women (bilateral hip arthroplasty).

d PDFF femoral neck measurements were not available in 4 women (bilateral hip arthroplasty, n=3; unacceptable quality of measurements, n=1).

Bold values correspond to significant results (p-value < 0.05).

Body composition parameters were comparable in both groups, except for total lean mass, which was higher in the non-fractured group compared to fractured women (36.5 kg (IQR: 33.4 to 39.1) versus 33.8 kg (IQR: 31.2 to 36.1), p=0.038).

Non-fractured postmenopausal women had significantly higher BMD at the lumbar spine, femoral neck, and total hip than in the fractured group (p<0.05 for all). Regarding bone marrow PDFF, no significant differences were found between those with and without fracture (**Table 1**). PDFF at the lumbar spine was higher in the fractured group than in non-fractured subjects but did not reach statistical significance (58.3 ± 10.2% versus 54.9 ± 9.8%, p=0.091).

### 3.2 PDFF measurements of the paravertebral muscles

The mean PDFF of the psoas and paravertebral muscles was higher in the fracture group than in non-fractured patients, even after adjustment for age, weight, and height (PDFF_Psoas_: 17.1 ± 6.1% versus 13.5 ± 4.9%, p=0.004, and PDFF_Paravertebral_: 34.4 ± 13.6% versus 24.9 ± 8.8%, p=0.002). No significant differences between fractured and non-fractured participants could be detected in CSA and CMI values of the psoas and paravertebral muscles after adjustment for age, weight, and height (**Table 2**).

**Table 2:** Paravertebral muscles of postmenopausal women.

	Fracture (n=56)	No fracture (n=46)	*p*-value	Adjusted *p-*value*
**PDFF [%]** **– Psoas**	17.1 ± 6.1	13.5 ± 4.9	**0.002**	**0.004**
**PDFF [%]– Paravertebral muscles°**	34.4 ± 13.6	24.9 ± 8.8	**<0.001**	**0.002**
**CSA [mm²]** **– Psoas**	689.8 ± 180.2	754.2 ± 186.2	0.080	0.63
**CSA [mm²]** **– Paravertebral muscles°**	1731.7 ± 359.9	1806.2 ± 312.0	0.27	0.42
**CMI [mm²]** **– Psoas**	568.9 ± 142.4	650.7 ± 160.5	**0.008**	0.22
**CMI [mm²]** **– Paravertebral muscles°**	1130.2 ± 308.9	1356.7 ± 287.0	**<0.001**	0.11

Values are expressed as mean ± SD. Statistically significant differences (p-value < 0.05) are in bold.

* adjusted for age, weight, and height.

°Posterior paravertebral muscles comprised the erector spinae and multifidus muscles.

PDFF: Proton Density Fat Fraction; CSA: Contractile Surface Area; CMI: Contractile Mass Index; SD: Standard Deviation.

### 3.3 Relationship between myosteatosis and bone marrow adiposity

In the fracture group, there was no significant relationship between PDFF in the psoas or paravertebral muscles and marrow adiposity, either at the lumbar spine or the femoral neck (**Table 3**). Surprisingly, in the non-fractured group, we found a significant relationship between higher PDFF_Paravertebral_ and lower PDFF at the lumbar spine (*β* = -6.80 ± 2.85, p=0.022) after adjustment for age, weight, height, and lumbar spine BMD. Expressed otherwise, each standard deviation increase in PDFF_Paravertebral_ was associated with lower PDFF lumbar spine (-6.8%).

**Table 3:** Relationship between Myosteatosis and Bone Marrow Adiposity .

	Model 1		Model 2		Model 3	
	β ± SE	*p*	β ± SE	*p*	β ± SE	*p*
PDFF at the lumbar spine						
FRACTURE (n=56)						
**PDFF Psoas**	0.58 ± 1.32	0.66	-0.28 ± 1.40	0.84	-1.06 ± 1.89	0.58
**PDFF Paravertebral muscles°**	0.96 ± 1.27	0.46	-0.58 ± 1.59	0.71	-1.13 ± 2.01	0.58
NO FRACTURE (n=46)						
**PDFF Psoas**	3.20 ± 1.70	0.066	1.33 ± 1.78	0.46	2.08 ± 2.10	0.33
**PDFF Paravertebral muscles°**	0.95 ± 2.12	0.66	-6.08 ± 2.54	**0.021**	-6.80 ± 2.85	**0.022**
PDFF at the femoral neck						
FRACTURE (n=56)						
**PDFF Psoas**	0.31 ± 1.09	0.78	-0.72 ± 1.10	0.52	1.43 ± 1.44	0.33
**PDFF Paravertebral muscles°**	-0.03 ± 1.12	0.98	-2.30 ± 1.24	0.070	-0.97 ± 1.55	0.53
NO FRACTURE (n=46)						
**PDFF Psoas**	0.35 ± 1.39	0.80	-0.99 ± 1.48	0.51	0.72 ± 1.62	0.66
**PDFF Paravertebral muscles°**	0.73 ± 1.67	0.66	-2.89 ± 2.20	0.19	-1.10 ± 2.33	0.64

β were calculated for a standard deviation increase of each parameter. Statistically significant models (p-value < 0.05) are in bold.

°Posterior paravertebral muscles comprised the erector spinae and multifidus muscles.

Model 1: without adjustment.

Model 2: adjusted for age.

Model 3: adjusted for age, weight, height, and bone mineral density.

SE, Standard Error; SD, Standard Deviation.

### 3.4 Relationship between myosteatosis and other fat depots

In both groups, there was a significant relationship between higher PDFF_Psoas_ and higher VAT after adjustment for age, weight, and height (*β* = 20.27 ± 9.62, p=0.040 in the fracture group, and *β* = 37.49 ± 8.65, p<0.001 in the non-fractured group) (**Table 4**). Each SD increase in PDFF_Psoas_ was associated with higher VAT (20.27 cm² and 37.49 cm², respectively).

**Table 4:** Relationship between myosteatosis, visceral adipose tissue, and total body fat .

	Model 1		Model 2		Model 3	
	β ± SE	*p*	β ± SE	*p*	β ± SE	*p*
Visceral adipose tissue						
FRACTURE (n=56)						
**PDFF Psoas**	44.28 ± 8.82	**<0.001**	51.08 ± 9.19	**<0.001**	20.27 ± 9.62	**0.040**
**PDFF Paravertebral muscles°**	37.23 ± 9.80	**<0.001**	54.86 ± 11.17	**<0.001**	16.87 ± 10.61	0.12
NO FRACTURE (n=46)						
**PDFF Psoas**	63.50 ± 12.17	**<0.001**	69.08 ± 13.48	**<0.001**	37.49 ± 8.65	**<0.001**
**PDFF Paravertebral muscles°**	47.35 ± 17.22	**0.009**	64.34 ± 23.84	**0.010**	13.30 ± 15.27	0.39
Total body fat						
FRACTURE (n=56)						
**PDFF Psoas**	5.52 ± 1.28	**<0.001**	7.26 ± 1.21	**<0.001**	0.59 ± 0.53	0.27
**PDFF Paravertebral muscles°**	3.78 ± 1.44	**0.012**	7.40 ± 1.52	**<0.001**	-0.19 ± 0.58	0.74
NO FRACTURE (n=46)						
**PDFF Psoas**	6.10 ± 1.90	**0.003**	6.78 ± 2.12	**0.003**	1.83 ± 1.37	0.19
**PDFF Paravertebral muscles°**	7.87 ± 2.25	**0.001**	12.98 ± 2.95	**<0.001**	6.57 ± 1.80	**<0.001**

β were calculated for a standard deviation increase of each parameter. Statistically significant models (p-value < 0.05) are in bold.

°Posterior paravertebral muscles comprised the erector spinae and multifidus muscles.

Model 1: without adjustment.

Model 2: adjusted for age.

Model 3: adjusted for age, weight, and height.

SE = Standard Error; SD = Standard Deviation.

In the non-fractured group, there was a significant relationship between higher PDFF_Paravertebral_ and higher TBF after adjustment for age, weight, and height (*β* = 6.57 ± 1.80, p<0.001). Each SD increase in PDFF_Paravertebral_ was associated with higher TBF (+6.57 kg).

### 3.5 Relationship between bone marrow adiposity and other fat depots

After adjusting for age, weight, height, and BMD in both groups, there was no significant relationship between PDFF lumbar spine and femoral neck with other fat depots (**Table 5**).

**Table 5:** Relationship between marrow adiposity, visceral adipose tissue, and total body fat .

	Model 1		Model 2		Model 3	
	β ± SE	*p*	β ± SE	*p*	β ± SE	*p*
Visceral adipose tissue						
FRACTURE (n=56)						
**PDFF Lumbar spine**	0.32 ± 11.37	0.98	0.36 ± 11.65	0.98	-0.54 ± 7.80	0.94
**PDFF Femoral neck**	-22.89 ± 10.82	**0.039**	-26.29 ± 11.57	**0.028**	-6.78 ± 8.42	0.42
NO FRACTURE (n=46)						
**PDFF Lumbar spine**	12.05 ± 13.22	0.37	7.33 ± 14.64	0.62	14.14 ± 7.75	0.076
**PDFF Femoral neck**	-29.48 ± 12.61	**0.024**	-36.81 ± 12.78	**0.006**	-12.62 ± 7.89	0.12
Total body fat						
FRACTURE (n=56)						
**PDFF Lumbar spine**	0.05 ± 1.57	0.98	0.46 ± 1.58	0.77	0.21 ± 0.41	0.60
**PDFF Femoral neck**	-3.76 ± 1.46	**0.013**	-3.43 ± 1.57	**0.034**	0.02 ± 0.44	0.97
NO FRACTURE (n=46)						
**PDFF Lumbar spine**	-1.42 ± 1.81	0.44	-2.31 ± 1.99	0.25	-1.32 ± 1.09	0.24
**PDFF Femoral neck**	-3.44 ± 1.75	0.056	-4.12 ± 1.82	**0.028**	-0.70 ± 1.12	0.53

β were calculated for a standard deviation increase of each parameter. Statistically significant models (p-value < 0.05) are in bold.

Model 1: without adjustment.

Model 2: adjusted for age.

Model 3: adjusted for age, weight, height, and bone mineral density.

SE, Standard Error; SD, Standard Deviation.

### 3.6 Repeatability

Intraclass correlation coefficients were excellent (over 0.90) for PDFF and CMI measurements in the psoas and paravertebral muscles. Intraobserver agreement was good for CSA measurements in the psoas muscles (0.89, with a 95% confidence interval of [0.78-0.95]) and excellent for the interobserver agreement (0.95, with a 95% confidence interval of [0.91-0.98]). Similarly, interobserver agreement was good for CSA measurements in the paravertebral muscles (0.89, with a 95% confidence interval of [0.79-0.95]) and excellent for intraobserver agreement (0.96, with a 95% confidence interval of [0.91-0.98]).

## 4 Discussion

**Figure 4:**
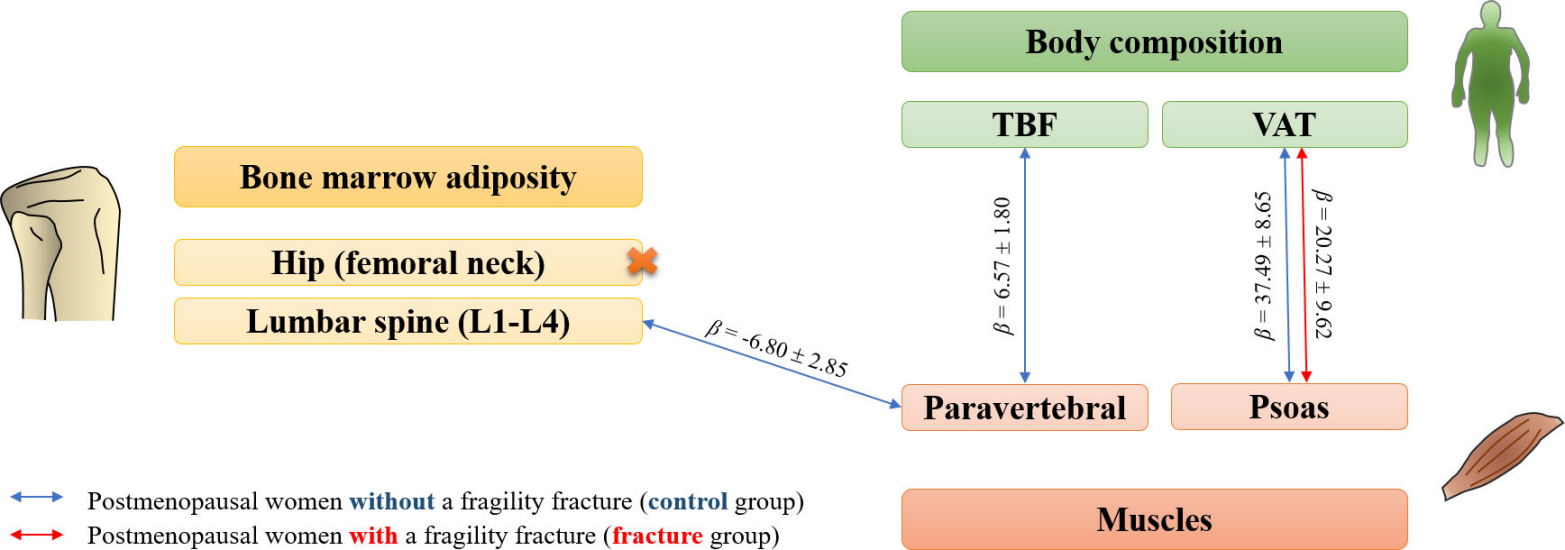
Diagram summarizing significant associations (*p*-value<0.05) between bone marrow adiposity, body composition and myosteatosis parameters in postmenopausal women with (red arrows) or without (blue arrows) a fragility fracture. Beta coefficients were extracted from regression models adjusted for age, weight and height, as well as body mineral density when considering bone marrow adiposity.

Using chemical shift encoding-based water-fat imaging, we investigated the complex relationship between bone marrow adiposity, myosteatosis, and other fat depots (summarized in **Figure 4**). The originality of our work lies in its design, comparing prospectively included postmenopausal women with and without a fragility fracture. Despite a higher intramuscular fatty infiltration in the fracture group, no relationship between bone marrow adiposity and myosteatosis was observed among patients with fragility fractures. On the contrary, but solely among postmenopausal women without fracture, we underlined an inverse interrelation between paravertebral myosteatosis and bone marrow adiposity at the lumbar spine, adjusted for age, weight, height, and lumbar BMD. Moreover, whereas relationships between myosteatosis, VAT, and TBF have been found, no significant association was underlined between bone marrow adiposity and other fat depots.

### 4.1 Myosteatosis and bone marrow adiposity

Fatty infiltration of multifidus and erector spinae muscles is well known to precede their atrophy after vertebral fracture, with no CSA change six months from onset (15). Accordingly, we observed higher fat deposits in paravertebral muscles among patients with a recent fragility fracture compared to controls without significant changes in CSA. This natural course highly suggests bone-muscle-fat interactions, supported by previous research on postmenopausal women with osteoporosis. The AMBERS cohort is indicative of this hypothesis. Using MRI and pQCT, this cross-sectional study reported that a higher amount of fat within muscles was associated with higher bone marrow adiposity at the mid-tibia, adjusting for age, weight, height, average daily energy expenditure, hypertension, and diabetes (7). However, no adjustment for BMD was performed, whereas a robust negative correlation has been observed with bone marrow adiposity (16–18). Our analysis considered BMD a potential confounder and did not reveal a relationship between bone marrow adiposity and myosteatosis in patients with fragility fractures. Interestingly, a higher amount of fat within the paravertebral muscles (erector spinae and multifidus) was associated with a lower PDFF at the lumbar spine among non-fractured women, adjusted for age, weight, height, and lumbar BMD.

The underlying mechanism explaining the differential effect between groups is unclear. The decreased physical activity with aging and muscle disuse makes the erector spinae and multifidus more vulnerable to fat infiltration than the psoas and lower limb muscles (19). A similar observation was reported in healthy men, with an increase in intermuscular adipose tissue of erector spinae but not psoas muscles with aging (20). In patients with a recent fragility fracture, one hypothesis is that the trends in a higher bone fatty infiltration compared to controls might mask the potential but weak bone-muscle interaction. Nevertheless, this novel finding supports an existing but fragile interaction between bone marrow adiposity at the lumbar spine and paravertebral myosteatosis in the absence of fragility fracture, with numerous potential actors at the cellular and hormonal levels (1).

### 4.2 Myosteatosis and other fat depots

Contrary to the underestimated importance of bone marrow adiposity, the role of ectopic fat infiltration into muscle –i.e., myosteatosis– has gained increasing attention in the scientific literature. Miljkovic et al. reported an independent association between abdominal myosteatosis (all muscles of the abdominal wall), hyperinsulinemia, and insulin resistance, even after adjusting for lifestyle factors, TBF, and visceral or subcutaneous adipose tissues (21). Indeed, skeletal muscle plays a crucial role in insulin resistance and may contribute to developing type 2 diabetes mellitus (22).

Furthermore, a strong link exists between myosteatosis and body fat. We observed that fatty infiltration in psoas muscles increased in patients with more extensive VAT, even after adjusting for age, weight, and height. Among postmenopausal women without fragility fractures, an association was found between PDFF in the erector spinae and multifidus muscles and TBF, based on the same adjustments. Although conducted in a cohort comprising only men over 65, these observations comply with the work from Miljkovic et al., reporting a relationship between psoas or paraspinal intermuscular adipose tissue with TBF and VAT (21). This proximity between muscle and visceral adipose tissues is supported by fundamental studies that observed similar molecular profiles (23). However, a more nuanced picture was depicted by Correa-de-Araujo et al., the association between myosteatosis and metabolic disorders being unclear when adjusting for visceral and ectopic fat depots (24). Nonetheless, our data suggest that skeletal and visceral fat depots share close functions from a metabolic perspective.

### 4.3 Bone marrow adiposity, a unique fat depot

Bone marrow adiposity is a recently recognized tissue, underestimated for a long time. Its peculiar relationship with skeletal health is best described by the paradoxical accumulation despite the depletion of other fat tissues in women with anorexia nervosa (25). Bone marrow adiposity may contribute to vertebral bone weakness and is inversely correlated with lower trabecular BMD in older women (26, 27). Potential clinical implications have been reported, but data are still scarce or inconsistent regarding its relationship with other fat depots (3).

Our analysis did not highlight any significant relationship between lumbar or femoral neck bone marrow adiposity and other fat depots after age, weight, height, and BMD adjustment. In a study based on a cohort of premenopausal women, Bredella et al. reported a positive correlation between vertebral bone marrow adiposity and VAT, even after adjusting for BMD (28). In patients with anorexia nervosa, an inverse correlation between the VAT and bone marrow adiposity at the vertebral level or proximal femur was observed without reaching significance (25). These inconsistencies in the investigated relationship between bone marrow adiposity and other fat depots suggest that bone marrow adiposity is uniquely regulated, with differential tissue connections depending on associated disorders such as obesity, anorexia nervosa, or osteoporosis.

### 4.4 Strengths and limitations

Our study comprises several strengths. **(1)** This ancillary work of the ADIMOS cohort yielded an original sample comprising prospectively included only postmenopausal women with or without a recent fragility fracture. This population is, therefore, homogeneous, limiting biases secondary to estrogen contribution. **(2)** Chemical shift encoding-based water-fat imaging was performed, considering the T1 bias and T2* decay, providing an accurate PDFF at the lumbar spine and paravertebral muscles (29). Despite the superior accuracy of MR spectroscopy, the use of chemical shift-based water-fat imaging allowed straightforward measurements achievable on most MR clinical systems while maintaining sufficient accurateness. Its main drawback was the lack of information provided about fat composition. Furthermore, although pQCT would bring more precise data on bone microarchitecture, MRI is considered as the gold standard and was preferred in our study to allow the exploration of the axial skeleton (30). Finally, DXA and MRI examinations were acquired on the same machines with the same parameters, reinforcing the comparability of the subjects’ data. **(3)** Literature is scarce on fat depots comparison. Our original approach compared myosteatosis with bone marrow adiposity in addition to conventional white adipose tissues (i.e., TBF and VAT). **(4)** Due to the strong correlation between bone marrow adiposity and BMD, our statistical model included this latter parameter to assess the relationship between the different fat depots independently.

However, a few limitations are also inherent to the study design and participants’ characteristics. **(1)** Although prospectively included, this study’s cross-sectional design and ancillary aspect restrained the temporal control between the occurrence of a vertebral fracture and imaging acquisitions. Nevertheless, the inclusion criteria outlined that only participants with a recent fracture (within 12 months) could be included. **(2)** Age and body parameters (weight and height) differed between groups. However, we adjusted for these characteristics in each statistical model we performed. Emergent anthropometric indices may be considered in future studies, such as the subcutaneous fat index (31). **(3)** Only a manual segmentation was achieved to quantify bone marrow adiposity and myosteatosis based on selected slices from MRI acquisitions. Semi-automatic or fully automated segmentation of vertebrae or paravertebral muscles would provide volumic features. However, we applied previously published methodological recommendations for assessing intramuscular fatty infiltration of paravertebral muscles (12). We also included the contractile mass index parameter (CMI) in our analyses to depict better the lean mass of the psoas, erector spinae, and multifidus muscles (9). Ultimately, intra- and interobserver agreements were good to excellent for MRI segmentation in our sample.

## 5 Conclusion

Using chemical shift encoding-based water-fat imaging, we demonstrated that bone marrow adiposity and myosteatosis were not associated among postmenopausal women with a history of fragility fracture, despite adjustments for age, weight, height, and BMD. Interestingly, an inverse association between fatty infiltration in the paravertebral muscles (erector spinae and multifidus) but not in the psoas was associated with bone marrow adiposity at the lumbar spine among controls. Our data also highlight bone marrow adiposity as a unique fat depot with a weak relationship to other white adipose tissues that may depend on the subjects’ comorbidities. Further research is needed to apprehend better biochemical regulations that affect this distinctive tissue, whose involvement in vertebral strength is underestimated.

## Data availability statement

The raw data supporting the conclusions of this article will be made available by the authors, without undue reservation.

## Ethics statement

The studies involving human participants were reviewed and approved by the Local Institutional Review Board (CHU Lille). The patients/participants provided their written informed consent to participate in this study.

## Author contributions

SB – Conceptualization, Methodology, Validation, Investigation, Writing (original), Writing (review), Supervision. HD – Validation, Data curation, Writing (review), Visualization. DL – Investigation, Resources, Data curation. HK – Validation, Data curation, Writing (review). CM – Methodology, Formal analysis, Writing (review). BC – Conceptualization, Resources, Writing (review), Project administration. AC – Writing (review). JP – Conceptualization, Methodology, Investigation, Resources, Writing (original), Supervision, Project administration, Funding acquisition. All authors contributed to the article and approved the submitted version.
